# Impact of respiratory therapy in vital capacity and functionality of patients undergoing abdominal surgery

**DOI:** 10.1590/S1679-45082016AO3398

**Published:** 2016

**Authors:** Shanlley Cristina da Silva Fernandes, Rafaella Souza dos Santos, Erica Albanez Giovanetti, Corinne Taniguchi, Cilene Saghabi de Medeiros Silva, Raquel Afonso Caserta Eid, Karina Tavares Timenetsky, Denise Carnieli-Cazati

**Affiliations:** 1 Hospital Israelita Albert Einstein, São Paulo, SP, Brazil.

**Keywords:** Breathing exercises, Respiratory function tests, Respiratory muscles, Vital capacity

## Abstract

**Objective:**

To evaluate the vital capacity after two chest therapy techniques in patients undergoing abdominal surgical.

**Methods:**

A prospective randomized study carried out with patients admitted to the Intensive Care Unit after abdominal surgery. We checked vital capacity, muscular strength using the Medical Research Council scale, and functionality with the Functional Independence Measure the first time the patient was breathing spontaneously (D1), and also upon discharge from the Intensive Care Unit (Ddis). Between D1 and Ddis, respiratory therapy was carried out according to the randomized group.

**Results:**

We included 38 patients, 20 randomized to Positive Intermittent Pressure Group and 18 to Volumetric Incentive Spirometer Group. There was no significant gain related to vital capacity of D1 and Ddis of Positive Intermittent Pressure Group (mean 1,410mL±547.2 *versus* 1,809mL±692.3; p=0.979), as in the Volumetric Incentive Spirometer Group (1,408.3mL±419.1 *versus* 1,838.8mL±621.3; p=0.889). We observed a significant improvement in vital capacity in D1 (p<0.001) and Ddis (p<0.001) and in the Functional Independence Measure (p<0.001) after respiratory therapy. The vital capacity improvement was not associated with gain of muscle strength.

**Conclusion:**

Chest therapy, with positive pressure and volumetric incentive spirometer, was effective in improving vital capacity of patients submitted to abdominal surgery.

## RESUMO

**Objetivo:**

Avaliar a capacidade vital comparando duas técnicas de fisioterapia respiratória em pacientes submetidos à cirurgia abdominal.

**Métodos:**

Estudo prospectivo e randomizado realizado com pacientes admitidos em Unidade de Terapia Intensiva após cirurgia abdominal. Verificamos a capacidade vital, a força muscular por meio da escala do *Medical Research Council* e funcionalidade pela Medida de Independência Funcional no primeiro momento em que o paciente encontrava-se em respiração espontânea (D1) e na alta da Unidade de Terapia Intensiva (Dalta). Entre D1 e Dalta, foi realizada a fisioterapia respiratória, conforme o grupo randomizado.

**Resultados:**

Foram incluídos 38 pacientes, sendo 20 randomizados para Grupo Pressão Positiva Intermitente e 18 para o Grupo Incentivador Inspiratório a Volume. A capacidade vital entre o D1 e Dalta do Grupo Pressão Positiva Intermitente não teve ganho significativo (média de 1.410mL±547,2 *versus* 1.809mL±692,3; p=0,979), assim como no Grupo Incentivador Inspiratório a Volume (1.408,3mL±419,1 *versus* 1.838,8mL±621,3; p=0,889). Houve melhora significativa da capacidade vital no D1 (p<0,001) e na Dalta (p<0,001) e da Medida de Independência Funcional (p<0,001) após a fisioterapia respiratória. A melhora da capacidade vital não apresentou relação com o ganho da força muscular.

**Conclusão:**

A fisioterapia respiratória, por meio de pressão positiva ou de incentivador inspiratório a volume, foi eficaz na melhora da capacidade vital em pacientes submetidos à cirurgia abdominal.

## INTRODUCTION

Static lung volume measurement plays an important role in pulmonary functional assessment, indirectly providing lung elasticity, and establishing forced expiratory flows.^(1)^ Abdominal surgery, be it upper (above the umbilical line) or lower (below the umbilical line), leads to changes in respiratory mechanics, lung volume and capacity, oxygenation, and pulmonary defense mechanisms. Shallow breathing occurs as a result of pain, reducing lung volume and capacity, which may last for 7 to 14 days after the surgical procedure.^(2,3)^

Respiratory therapy is helpful from the prevention to the treatment of pulmonary complications and comprises several techniques.^(4)^ These techniques are clinically significant and widely used because they increase functional residual capacity, ensure greater alveolar stability, and may be executed with or without mechanical devices.^(5)^

The objective of using of the incentive spirometer is to encourage the patient, through visual feedback, to sustain maximum inspiration.^(6,7)^ Despite the widespread use of the incentive spirometry, some systematic reviews suggested that this technique shows little evidence of benefits in the prevention of postoperative complications.^(4)^

Bi-level positive airway pressure, whether continuous or intermittent, has proven beneficial in prevention and treatment of pulmonary complications after heart surgeries.^(8)^ There are technical differences between bi-level positive continuous and intermittent airway pressures, because each of them acts in a specific way in the recovery of pulmonary function and respiratory mechanics. A review study showed the efficacy of continuous positive airway pressure, aiming to reduce the risk of pulmonary complications in patients undergoing abdominal surgery.^(9)^

However, the efficacy of respiratory therapy during postoperative abdominal surgery is still controversial. Pasquina et al.^(10)^ suggested that using routine respiratory therapy is unjustified, since few clinical trials show its effectiveness in prophylactic treatment. Nevertheless, Lawrence et al.^(11)^ stated that, in postoperative period of abdominal surgery, any pulmonary expansion technique is better than no prophylaxis.

It is known that respiratory therapy plays an important role in pulmonary rehabilitation, regardless of the technique employed. However, there are few studies that significantly express a comparison between techniques, such as the incentive spirometer and bi-level intermittent positive airway pressure during bedside patient care in the postoperative period of abdominal surgery (upper or lower).

## OBJECTIVE

To assess vital capacity by comparing two respiratory therapy techniques in patients undergoing abdominal surgery.

## METHODS

A randomized prospective analysis, in patients admitted to the adult and clinical-surgical Intensive Care Unit (ICU), in a private hospital. This study was approved by the Research Ethics Committee, under protocol number 214.411, CAAE: 12309513.6.0000.0071.

Patients included were over 18 years of age and underwent abdominal surgery, with or without pulmonary complications. We excluded patients with hemodynamic instability, previously diagnosed respiratory and/or neuromuscular diseases, and those who were uncooperative with physical therapy care.

Randomization was done by a draw to divide the patients into two groups: Positive Intermittent Pressure Group and Volumetric Incentive Spirometer Group.

Measurement of vital capacity (VC) was performed as described by the American Thoracic Society and by the European Respiratory Society,^(12)^ on the first day the patient started breathing spontaneously (D1) and on the day of discharge from the ICU (Ddis). Vital capacity was always checked at the beginning of treatment (D1 before and Ddis before) and at the end of respiratory therapy (D1 after and Ddis after) and 30 minutes after it (D1-30 and Ddis-30). Between days D1 and Ddis, the patients underwent conventional physiotherapy, as part of the institutional routine of the ICU physical therapy team, with lower limb free or assisted active exercises (according to the patient’s condition), respiratory physiotherapy associated to upper limb free or assisted active exercises, assisted cough, and, if necessary, nasotracheal aspiration for bronchial hygiene. The respiratory therapy technique to be used with patients, according to the randomized group, was followed at all times.

Later, muscle strength was assessed through the Medical Research Council (MRC) scale^(13-15)^ and through the Functional Independence Measure (FIM) indicator. These measurements were collected on D1 and Ddis.

With regards to respiratory therapy at the time of patient assessment in the study, the following exercises were performed: lower limb free or assisted active exercises (according to the patient’s condition); respiratory physiotherapy associated to upper limb free or assisted active exercises, assisted cough, and, if necessary, nasotracheal aspiration.

To wrap up the treatment according to randomization, bi-level intermittent positive airway pressure was performed in the Positive Intermittent Pressure Group, with the proper equipment for non-invasive, bi-level pressure mechanical ventilation, with inspiratory airway pressure and end-expiratory airway pressure. The (Ventilator iSleep, Breas^®^, Mölnlycke, Sweden), was used in three series of ten repetitions and with pressures determined for each patient, according to their ideal tidal volume and following the mechanical ventilation consensus guidelines (tidal volume of 6mL/kg), in accordance with equipment monitoring.

Volumetric incentive spirometer (Voldyne 5000, Hudson RCI^®^, Tecate, México), was used by the Volumetric Incentive Spirometer Group, in three series of ten repetitions each.

Due to the lack of studies showing the benefits between the use of bi-level intermittent positive airway pressure techniques and volumetric incentive spirometer in postoperative patients who underwent abdominal surgery, it was possible to do the calculation of the sample based on the results of the first 10 cases assessed (pilot). Vital capacity variability was shown at 30 minutes of approximately 650mL, where there was an assumed difference of 600mL between the groups, with 80% power and 95%IC. Thus, the required sample for the study was 19 patients per group.

Qualitative personal characteristics were described according to the groups, through absolute and relative frequencies, and the association between them was verified using χ^2^ or Fisher’s exact tests, which was also used when the sample was insufficient for χ^2^ test.

Quantitative personal characteristics were described according to the groups through summary measures (mean, standard deviation, median, minimum and maximum), and compared amongst each other by Student’s *t* test or Mann-Whitney, in the absence of normal distribution of the variable, which was evaluated through Kolmogorov-Smirnov test.

Vital capacity results were described on the second day of assessment and at the moments to evaluate measurements during therapy (before, at the end of therapy, and 30 minutes after). MRC and FIM results were described as days of assessment and using summary measurements (mean, standard deviation, median, minimum and maximum).

The correlation between VC and MRC and FIM functionality scores was assessed in both groups, by calculating Pearson’s correlations.

Tests were performed with a significance level of 5%. Statistical analysis was done through the software Statistical Package for Social Sciences (SPSS), version 13.0.

## RESULTS

The study comprised a sample of 38 individuals who were divided into two groups after randomization. The Positive Intermittent Pressure Group was formed by 20 individuals, and the Volumetric Incentive Spirometer Group by 18 individuals. The sample did not show significant difference between age, total days at the ICU, and total days of hospital stay between the groups.

The groups were homogenous and did not show significant difference between their characteristics, such as gender, personal background, and respiratory complications. The only significant difference was in the fact that the Positive Intermittent Pressure Group showed a higher number of upper abdominal surgeries (80%), and the Volumetric Incentive Spirometer Group showed a higher number of lower abdominal surgeries (83.3%) (p<0.001). However, the type of abdominal surgery was not a criterion for randomization interference (Table 1).

**Table 1:** Description of the sample

Variables	Positive Intermittent Pressure Group	Volumetric Incentive Spirometer Group	p value
Age (years),* mean (SD)	58.70 (15.9)	63.67 (15.6)	0.340
Gender^†^ – n (%)			0.703
Female	9 (45)	7 (38.9)	
Male	11 (55)	11 (61.1)	
Smoking^†^ – n (%)	1 (5)	1 (5.6)	>0.999
Past history^†^ – n (%)			
Hypertension	4 (20)	8 (44.4)	0.106
DM	4 (20)	5 (27.8)	0.709
Type of abdominal surgery^†^ – n (%)			<0.001
Higher	16 (80)	3 (16.7)	
Lower	4 (20)	15 (83.3)	
Respiratory complications^†^ –n (%)			>0.999
Atelectasis	3 (15)	0 (0)	0.232
Pleural effusion	3 (15)	0 (0)	0.232
Length of stay at ICU (days)^‡^ – median (max-min)	2.75 (1-7)	2.44 (1-4)	0.696
Length of stay (days)^‡^ – median (max-min)	10 (5-88)	12 (5-27)	0.497

*Student´s *t* test. ^†^χ^^2^^. ^‡^Mann-Whitney test.

SD: standard deviation; DM: *diabetes mellitus*; ICU: intensive care unit.

**Figure 1:**
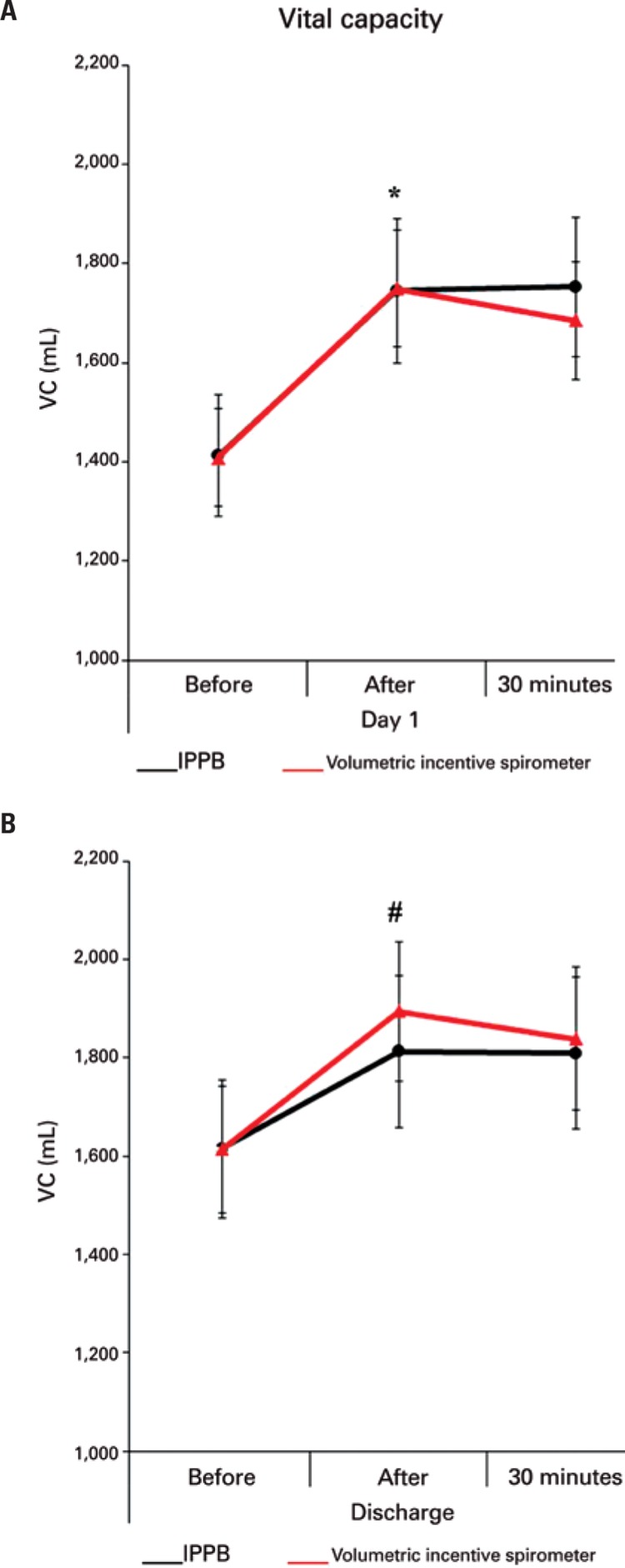
Vital capacity measurements in each (D1) and (Ddis) of the patient. (A) Vital capacity measurements in the first therapy session. (B) Vital capacity measurements in the last therapy session at the Intensive Care Unit

Correlation of VC measurements between the Positive Intermittent Pressure Group and the Volumetric Incentive Spirometer Group did not show significant difference (p=0.969). However, in regards to the correlation between each moment, there was significant difference in VC measurements (Figure 1). There was significant gain between D1 before and D1 after (p<0.001) and D1 before and D1-30 (p<0.001). However, there was no significant difference between D1 after and D1-30 (p>0.999), or between D1-30 and Ddis before (p=0.308). There was significant VC improvement between Ddis before and Ddis after (p<0.001).

*Vital capacity was significantly higher after therapy than before, p<0.001. #Vital capacity was significantly higher after therapy than before, p<0.001.

VC: vital capacity; IPPB: intermittent positive pressure breathing.

After the surgical procedure, all patients presented VC below the predicted lower limit. Even with significant gain after respiratory therapy, VC measurements remained below the predicted lower limit^(1)^ (Table 2).

**Table 2:** Vital capacity measurement in the first (D1) and last (Ddis) therapy session at the Intensive Care Unit, and predicted lower limit of vital capacity in the Positive Intermittent Pressure Group and Volumetric Incentive Ipirometer Group

Variables	Positive Intermittent Pressure Group	Volumetric Incentive Spirometer Group	p value
D1 before*, mL – mean (SD)	1,412.50 (547.2)	1,408.33 (419.1)	0.979
Ddis 30*, mL – mean (SD)	1,809.00 (692.3)	1,838.89 (621.3)	0.889
Predicted lower limit VC*, mL – mean (SD)	3,094.35 (769.9)	2,789.33 (543.4)	0.171

*Student´s *t* test.

SD: standard deviation; VC: vital capacity.

**Figure 2:**
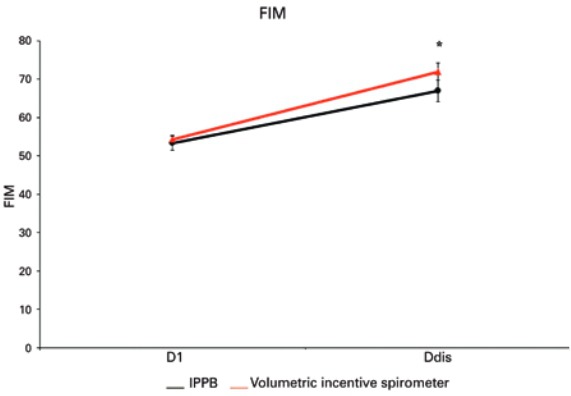
Functional Independence Measure performed on the first (D1) and last (Ddis) respiratory therapy session before discharge from the intensive care unit

**Figure 3:**
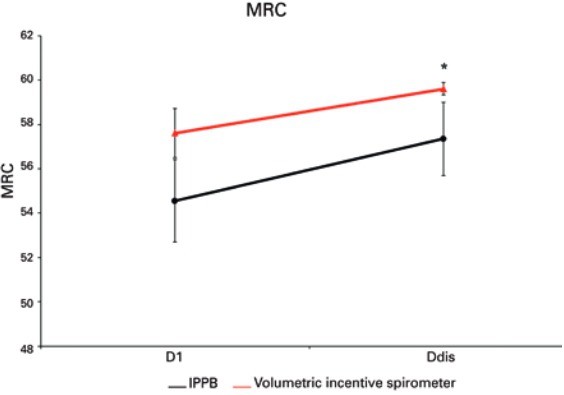
Assessment of overall muscle strength performed on the first (D1) and last (Ddis) respiratory therapy session before discharge from the intensive care unit

With regards to measurements of FIM (Figure 2) and MRC (Figure 3) scales, no significant difference was found between the two groups (p=0.204 and p=0.160, respectively). However, when comparing them between D1 and Ddis, a significant increase was found in both FIM (p<0.001) and MRC (p=0.003).

*Ddis is significantly higher than at D1, p<0.001.

FIM: functional independence measure; IPPB: intermittent positive pressure breathing.

*Ddis is significantly higher than at D1, p=0.003.

MRC: medical research council.; IPPB: intermittent positive pressure breathing.

No correlation was found between VC and functionality measurements FIM and muscle strength MRC, on D1 (r=-0.094 and r=0.211; p>0.05) or on Ddis (r=0.265 e r=0.189; p>0.05), respectively.

## DISCUSSION

Upper or lower abdominal surgery has a great impact on patients’ pulmonary mechanics, with a direct influence on lung capacity and volume.^(16)^ In our sample, all patients arrived at the ICU with VC below the predicted lower limit and were more prone to pulmonary complications. Nonetheless, even though these measurements were below the lower limit, there was significant gain in VC after respiratory therapy.

Respiratory therapy, with volumetric incentive spirometer or intermittent positive pressure, is efficient with regards to VC gain for patients undergoing abdominal surgery. In both the Positive Intermittent Pressure Group and the Volumetric Incentive Spirometer Group, VC gain was observed when comparing the first measurement before respiratory therapy and the last measurement before discharge from ICU.

The Positive Intermittent Pressure Group was formed by a higher percentage of upper abdominal surgeries, most of which were liver transplants, whereas the Volumetric Incentive Spirometer Group had a prevalence of lower abdominal surgeries. Although the groups did not present significant differences between them, literature shows that upper abdominal surgeries tend to trigger more pulmonary complications.^(2,3,10)^ However, in this study, neither group showed significant pulmonary complications.

Marques et al.^(17)^ stated that the volumetric incentive spirometer is better than the flow incentive spirometer, because it promotes a more efficient respiratory pattern, that is, a predominantly abdominal breathing with superior inspiratory time and reduced respiratory muscle overload, which brings more comfort to the patient and more efficiency during the exercise. Respiratory therapy is important in the ICU, and, among all the techniques used in that environment, intermittent positive pressure appears as a device that may help increase lung volumes and optimize gas exchange.^(18,19)^

Regardless of the technique or device here employed, there is evidence that respiratory therapy is beneficial and efficient when applied to bed-ridden patients with respiratory mechanics alterations triggered by surgical procedures.

In our results, the most significant gain happened on the first day of therapy, when we observed a significant difference between D1 before and D1 after. Between D1 after and D1-30, there was a small drop in VC, which was still higher than D1 before. This suggests that respiratory therapy in early postoperative stages may prevent VC from staying too low and, consequently, prevent other pulmonary complications. Despite a small decrease after therapy, there is still gain in pulmonary function. However, when comparing D1 to Ddis, the gain between the two moments was not as significant as on D1.

Our sample showed the sustained gain in VC, suggesting that respiratory therapy improves the patient’s VC and helps maintain that gain throughout time. This is an extremely important point, since these patients present with altered respiratory mechanics and need respiratory and functional re-education in the postoperative period.

In relation to patient functionality, rehabilitation was efficient in the gain of these measurements. Initially, the patients showed decreased overall muscle strength, with consequent restriction of daily functions and, after rehabilitation, overall muscle strength showed significant improvement. Therefore, in order to minimize risk of immobility caused by postoperative pain or complications, respiratory therapy promotes increase of muscle strength and improves functionality in activities of daily life.

Muscle strength and functionality are directly related. When a patient presents reduced muscle strength, decrease or loss of functionality occurs as a consequence.^(18)^ Correlating these two important items to pulmonary function, we observed that MRC and FIM gains did not have significant relation to VC. This suggests a gain in overall muscle strength with consequent improvement of patient’s functionality, but also that this improvement is not directly related VC improvement. This has us considering that the improvement in VC was related to respiratory function optimization with lung volume and capacity increase, and not to the gain of overall muscle strength.

Ferreira et al.^(20)^ stated that with respiratory therapy after abdominal surgeries, such as cholecystectomy, the patient presents improvements in respiratory mechanics and also gains in functionality and quality of life, due to muscle strength improvement and cardiorespiratory fitness.

A possible limitation of this study was due to the short ICU stay as a result of high patient turnover. However, patients undergoing abdominal surgery may present changes in ventilatory mechanics immediately after surgery, which prompts pulmonary complications. Another limitation was the authorization by the medical staff to use of positive pressure in the postoperative period; however the proposed goal was reached.

## CONCLUSION

Respiratory therapy done with bi-level intermittent positive airway pressure and/or volumetric incentive spirometer benefited the patients in the postoperative period of abdominal surgery, improving vital capacity. The was no correlation between vital capacity gain and the Functional Independence Measure and the Medical Research Council scales, but the patients presented increased strength and functionality separately. This suggested that respiratory therapy aids in overall muscle strength improvement, with enhanced functionality, bringing more independence to the patients in their activities of daily life.
